# Male infertility and the human microbiome

**DOI:** 10.3389/frph.2023.1166201

**Published:** 2023-06-09

**Authors:** Resa G. Magill, Susan M. MacDonald

**Affiliations:** ^1^ McGovern Medical School, University of Texas Health Science Center at Houston, Houston, TX, United States; ^2^ Department of Urology, The Pennsylvania State University College of Medicine, Hershey, PA, United States

**Keywords:** male infertility, microbiome, probiotics, andrology, testicular microbiota, oxidative stress, azoospermia

## Abstract

The historical belief in urology was that the genitourinary system should be sterile in a normal, healthy, asymptomatic adult. This idea was perpetuated for decades until research revealed a diverse microbiota existing in human anatomical niches that contributed to both human health and disease processes. In recent years, the search for an etiology and modifiable risk factors in infertility has turned to the human microbiome as well. Changes in the human gut microbiome have been associated with changes in systemic sex hormones and spermatogenesis. Certain microbial species are associated with higher levels of oxidative stress, which may contribute to an environment higher in oxidative reactive potential. Studies have demonstrated a link between increased oxidative reactive potential and abnormal semen parameters in infertile men. It has also been hypothesized that antioxidant probiotics may be able to correct an imbalance in the oxidative environment and improve male fertility, with promising results in small studies. Further, the sexual partner's microbiome may play a role as well; studies have demonstrated an overlap in the genitourinary microbiomes in sexually active couples that become more similar after intercourse. While the potential applications of the microbiome to male fertility is exciting, there is a need for larger studies with uniform microbial sequencing procedures to further expand this topic.

## Introduction

The Human Genome Project (HGP), launched in 1990, aimed to map the entire human genome, which led to significant advancements in our understanding of human biology and genetics (1). As part of this project, researchers also started to investigate the microbial communities that inhabit the human body and their role in human health (2). The microorganisms inside humans, known as the microbiota, outnumber human cells ten to one (3). The microbiota's genomes, collectively referred to as the microbiome, can provide traits and functions that humans did not evolve on their own, and produce a composition of small molecules and downstream products, referred to as the metabolome (3). If we are thought of as a composite of microbial and our own cells, then human health and disease develops from both genomes, the microbiome and our own (3). With advances in molecular biology techniques such as Next Generation Sequencing and 16S rRNA subunit analysis, scientists were able to characterize an entire microbial community in a sample, rather than just an individual species (4). These advances revealed that microbiomes exist in nearly every anatomical niche in the body, and seem to play a role beyond infection (3). The individuality of the microbiome and dynamic changes it undergoes in adulthood may allow an opportunity to treat disease on a more personalized level, and to understand variances in disease susceptibility and treatment response.

## Gut-testes axis

As demonstrated in other systems, there is a hypothesized link between the gut microbiome and the male urogenital tract. The gut-testes axis refers to an interaction between the microbiome of the gut and its impact on regulating testicular function, and is thought to play a role in male reproductive health and infertility. There is significant evidence that an alteration in the GM can lead to systemic changes and inflammation, thus may affect the testicular environment and form an interdependent connection (5). Trimethylamine N-oxide (TMAO) is thought to be a key signaling molecule. TMAO is a biologically active molecule generated by the GM and associated with an increased risk of atherosclerosis and endothelial dysfunction (6). High levels of TMAO are associated with fewer and less healthful endothelial progenitor cells and more reactive oxygen species, potentially contributing to vasculogenic erectile dysfunction (7).

A paper written by Tremellen in 2016 highlighted a potential mechanism that links gut dysfunction in obese men and late onset hypogonadism, formally referred to as “The Gut Endotoxin Leading to a Decline in Gonadal Function” (GELDING) theory (8). The theory states that a high fat, high calorie diet can trigger a breakdown in intestinal mucosal barrier, leading to a leakage of bacterial endotoxin and a chronic state of low grade inflammation (8). This state of low grade inflammation can then lead to testicular and hormonal dysfunction (8). There is evidence that a high fat diet can alter disordered tight junction proteins that separate the metabolome of gut microbes from leaking into systemic circulation (9). One study in male mice demonstrated the consumption of a high-fat diet altered the seminal fluid and gut microbiomes (10). This theory is also supported by data in animal studies that show certain endotoxins can impair luteinizing hormone (LH) pulses and interfere with intra-testicular production of testosterone (11, 12).

Several studies have demonstrated that the gut microbiome influences testosterone levels and sperm production. Bacterial fermentation produces certain short-chain fatty acids, which trigger the release of gut hormones such as GLP-1 and peptide YY (PYY), two anorexigenic peptides that regulate satiety (13, 14). GLP-1 agonists have also been shown to increase serum testosterone in diabetic and/or obese patients, improve sperm metabolism, motility, and insulin secretion in the testes (15). PYY administration inhibits GnRH secretion in male rats and delays the LH surge in female ewes (16, 17). The GM also directly generates androgens, thus changes in the GM may modulate intestinal and androgen metabolism (18). Additionally, androgen deprivation can alter fecal microbiota and exacerbate risks for other systemic diseases such as obesity and cardiovascular disease (19).

Animal studies have also shown that a FMT can treat infertility and improve sperm quality, demonstrating another link between gut dysbiosis and fertility. A study in mice by Zhang et al. looked at the effects of a FMT in mice with damaged spermatogenesis and sperm quality. The study found that a FMT improved spermatogenesis, increased sperm concentration, improved motility, increased levels of testicular antioxidants and key reproductive proteins, and increased gene expression related to spermatogenesis (20). In mice with induced Type 1 diabetes and infertility, a FMT significantly decreased blood glucose and increased semen quality, improved spleen and liver functions to strengthen the systemic environment for sperm development, and increased blood and testicular key metabolomes, such as docosahexaenoic acid (DHA) and testosterone (21). Other studies have demonstrated decreased DHA in the testes of animals with high fat diets, and supplemental DHA has been associated with restored fertility in infertile male mice (22, 23).

In addition to evidence from animal experiments, a few clinical trials have shown an association between the gut microbiome, serum testosterone levels, and sperm production (24–27). In an observational study of men with type 2 diabetes, significant differences in microbiota species were noted between men with and without low testosterone (26). Men with low serum testosterone also had an increased abundance of opportunistic pathogens (26). Another study demonstrated a significantly positive association between gut levels of *Firmicutes* and serum testosterone in adult men, while controlling for age, BMI and lipoprotein levels (28). There is further evidence through endocrine research that certain hormones involved in steroidogenesis are actively synthesized in the colon and under the influence of the GM (29–31). One study found that a specific microbial species found in the gut and genitourinary tract is capable of metabolizing both endogenous glucocorticoids and pharmaceutical derivatives, potentially influencing bioavailability of certain metabolites that may influence hypertension and prostate growth (29). Overall, the combination of these studies demonstrates a link between hormones and microbes, but more research especially in humans is needed in regards to male fertility specifically.

## Male infertility

While the urogenital microbiome shows similarity with the gastrointestinal microbiome in individuals, there are significant differences that suggest a distinct upper genital tract contribution (32). Studies have shown that the list of bacteria was shorter and the total count of bacteria was lower in first catch-urine (representing urethra microbiome) than in first-catch seminal fluid, suggesting a unique contribution of the upper genital tract (33). The diversity of seminal microbiome has been demonstrated with several studies, with one citing >10^4^ bacteria per ml in both healthy and infertile men, without any differences detected in semen parameters or semen specimens with and without bacteria (34). Analysis of 97 semen samples showed that 83% contained bacteria, and the presence of multiple bacterial species in semen was not associated with abnormal sperm function (35). The seminal vesicles, prostate, and testes have their own unique microbiomes, which differ between individuals and at different points in time, and can be altered based on individual hygiene, circumcision, sexual practices, sexual partners, and diet (36). For example, lower bacterial concentration and diversity were observed in men without sexual experience compared to those with sexual experience (37). In a healthy man, the most abundant species varies throughout the urogenital tract, which makes it difficult to fully understand what constitutes a healthy seminal microbiome (36).

There have been several studies that have looked at the role of seminal dysbiosis and its relation to prostatitis. While a number of microorganisms have been reported to cause chronic prostatitis, there is a growing hypothesis that prostatitis could be the result of seminal dysbiosis (38). Other studies have linked chronic prostatitis to inflammatory bowel syndrome, with simultaneous presence of syndromes occurring in about 30% of patients (39). The pathophysiology of these two simultaneous conditions is thought to be secondary to intestinal and or seminal dysbiosis and persistent mucosal inflammation (40). A study by Mandar et al. in 2017 revealed men with prostatitis had overall higher species richness and lower counts of *Lactobacilli* (41). Lactobacilli are associated with protective functions in oral cavity, vagina, and gastrointestinal tract, and may possibly play a similar role in the male reproductive tract (42). In studies of semen quality in infertility, >80% of normal semen samples clustered into a *Lactobacillus* predominant group (43).

The microbiome may be a tool to help improve male factor fertility. Estimates of idiopathic male infertility range from 30%–50%, citing underlying factors such as oxidative stress, lifestyle, and unidentifiable hormonal imbalances (44, 45). Oxidative stress (OS) from both exogenous (e.g., alcohol) and endogenous metabolic processes is a contributor to idiopathic male infertility in an estimated 80% of cases (44). Dysfunctional cellular processes contribute to the generation of reactive oxygen species (ROS) in semen, which in turn negatively impacts sperm function and membrane fluidity (46). An increase in oxidation-reduction potential, a measure of ROS, is associated with abnormal semen parameters in infertile men (47).

There has also been an established link between dysbiosis and increased oxidative stress; certain bacterial species may generate ROS through bacteriospermia, adhesion, toxin production, and inflammation (48). Studies of sperm quality and oxidative markers revealed that bacteriospermia is associated with inflammation, oxidative stress and sperm structural deterioration (49). Another study revealed that the intake of ultra-processed foods (UPFs) and a disruption of the normal GM was positively associated with the odds of asthenozoospermia (50). There is also an established link between gut dysbiosis and an increase in systemic oxidative stress potentially associated with other diseases such as Alzheimer's dementia, depression, and type 2 diabetes mellitus (51).

Since the establishment of a microbiome in the testis and semen, research has focused on trying to identify specific taxonomic correlations with infertility (36, 52). While studies have tried to find an association between the presence of seminal dysbiosis and infertility, the results are still conflicting (52, 53). Several studies conclude *Lactobacillus* is associated with a protective impact on semen parameters, yet *Ureaplasma urealyticum, Mycoplasma hominis,* and *Aerococcus* have been shown to have a negative impact on semen parameters. *Bacterioidetes* and *Firmicutes* are associated with azoospermatic semen (32, 43, 52–58). A recent study from Garcia-Segura in 2022 identified several bacteria strains in the seminal microbiome in infertile men that were negatively correlated with sperm global DNA fragmentation, specifically *Moraxella, Brevundimos,* and *Flavobacterium* (59). Other species correlated with reduced chromatin protamination status and increased in DNA fragmentation, negatively effective sperm reproductive potential (59). There is still a scarcity of data on this subject, with meta-analyses incorporating data from non-human subjects to supplement human trials, however, the data overall supports the hypothesis that certain bacterial strains in semen can alter the quality of sperm (53).

Although the link between male infertility and the taxonomy of the microbiome is unclear, the function of the microbiome may be more important (60). Despite differences in microbiota composition between individuals, their overall microbiome may perform the same functions, producing the same metabolites and causing the same changes in human cells (61). A study by Lundy et al. found that infertile men had significant alterations in the S-adenosyl-L-methionine (SAM) cycle overrepresented in their urine and semen (32). The SAM cycle is involved in DNA methylation, oxidative stress, and polyamine synthesis, however, its specific role in fertility is currently unknown (32). This study suggests that the specific metabolic processes that the microbiome performs, rather than its composition, may be affecting fertility. Further research is needed to better understand the functional processes of the microbiome and their association with infertility.

Another area for further exploration is the role of combined seminovaginal microbiome and its potential influence on the couples' fertility. Sexual intercourse can alter both the urinary and vaginal microbiomes in women, with studies showing an increased *G. vaginalis* in young women after sexual activity (62). There is additional data to suggest sexual intercourse can change the male urethral microbiome, with certain species such as *Ureaplasma, Mycoplasma* and *Sneathia* only found in sexually experiences males (63). While data around the microbiome and male infertility is rare, parallel studies examining the microbiota of sexually active couples are even rarer (64). A study done by Mandar et al. found major shifts in the vaginal microbiome after intercourse; a predominance of *G. vaginalis* in women was significantly associated with leukocytospermia in their male partners (64). Leukocytes in sperm are also the number one contributor to seminal ROS, potentially contributing to male infertility (44). In addition, Wittemer et al. demonstrated that positive bacterial cultures from both vagina and semen in couples undergoing *in vitro* fertilization decreased the clinical pregnancy rate and increased the spontaneous miscarriage rate significantly more than vaginal infection alone (65). The concept of olfactory receptors and volatile short-chain fatty acids (SCFAs) could provide the underlying mechanism for a seminovaginal microbial role in fertility. Olfactory receptors are key receptors involved in spermatozoa chemotaxis, and can be activated by SCFAs, metabolic by-products of anaerobic bacterial fermentation (66). A study by Teveroni et al. in 2022 demonstrated spermatozoa activation after olfactory receptors bound to SCFAs produced by microbiota metabolism in cervical mucus (66). This mechanism relied on a bacterial process in the vagina, thus providing a possible mechanism for a microbiome's role in a couple's fertility.

## Probiotics for male infertility

In the case of a disordered microbiome and excess ROS contributing to male infertility, probiotics and prebiotics with antioxidant properties may be a way to create an environment that is more favorable for spermatozoa. Probiotics contain live microorganisms, while prebiotics are food for human microbiota. There are seven core genera of organisms that are most often used in probiotics, those being *Lactobacillus, Bifidobacterium, Saccharomyces, Streptococcus, Enterococcus, Escherichia,* and *Bacillus* (67). Probiotics may be able to reset reproductive tract dysbiosis (68). Antioxidants have been shown to enhance the semen quality in smokers, prevent release of immature sperm, and have a positive impact on assisted reproductive technology (44). Oral probiotics have shown to be successful in treating female reproductive concerns, and thus have the potential to optimize the male microbiome as well (69, 70). A meta-analysis on probiotics for female reproductive tract concerns highlighted that oral probiotics can effectively restore vaginal microbiomes to optimal *Lactobacillus* spp dominance (71). An imbalance in this species has been associated with adverse pregnancy outcomes, a significant decrease in endometrial implantation, and altered IVF outcomes (71). The species also produces bacteriocins with antimicrobial activity against relevant urogenital pathogens (71). Some probiotic species can also improve immune responses, with one study demonstrating a reduction in endometrial lesions through endometrial epithelial cell barrier function with probiotic administration (72).

Certain studies have examined the use of probiotics couples with antibiotics in treating infertility associated with infections, which are outlined in Table 1. A study by Grande et al. treated 104 infertile patients with semen and/or prostatic secretions positive for gram-negative bacteria with fluoroquinolones, with 84 receiving probiotic *Enterococcus faecium* and *Saccharomyces boulardii*, followed with Lactobacilli, in addition to antibiotics (73). A negative semen culture was observed in 76.2% of patients in the combination treatment group, compared to only 50% in the antibiotics alone group (73). Another study looked at the progression of chronic bacterial prostatitis (CBP) in patients with infertility and concomitant inflammatory bowel disease after treatment with antibiotics (40). Patients were treated with a combination of rifaximin and a probiotic strain of eight gram-positive bacteria species (40). The men in the therapeutic treatment group had a lower risk of progression of prostatitis into prostate-vesiculitis than men given no treatment (40). These studies demonstrate a benefit to adding probiotics to antibiotic treatment when treating for potential infections that contribute to infertility.

**Table 1:** Examples of studies on the effects of probiotics and male reproductive health and fertility.

Test group	Probiotic species	Result
**Studies on humans**		
Infertile male patients with semen and/or prostatic secretions positive for gram-negative bacteria	*Enterococcus faecium, Saccharomyces boulardi, Lactobacilli*	Negative semen culture more likely in combined probiotic and antibiotic group (73)
Men with asthenozoospermia	*Lactobacillus rhamnosus CECT8361* and *Bifidobacterium longum CECT7347*	Improved sperm motility, decreased DNA fragmentation, decreased intracellular hydrogen peroxide, no change in cell viability (74)
Men with idiopathic oligoasthenoteratospermia	*Lactobacillus paracasei, arabinogalctan, oligo-fructosaccharides and l-glutamine*	Improved sperm count, sperm concentration, progressive motility, and percentage of typical forms in group treated with probiotics (75)
Men with idiopathic oligoasthenoteratozoospermia	*Lactobacillus casei, Lactobacillus rhamnosus, Lactobacillus bulgaricus, Lactobacillus acidophilus, Bifidobacterium breve, Bifidobacterium longum,* and *Streptococcus thermophil*	Improved serum testosterone, ejaculate volume, sperm concentration, total antioxidant capacity of plasma in men taking probiotics (76)
**Studies on animals**		
Zebrafish	*Lactobacillus rhamnosus* and *Bifidobacterium longum*	Increased sperm concentration, motility, and progressive sperm motility and treatment group (77)
Male broilers	*Bacillus amyloliquefaciens TOA5001*	Improved sperm concentration, improved activity of antioxidative associated enzymes (78)
Male mice	*Lactobacillus rhamnosus PB01*	Improved serum testosterone, LH, and FSH, more motile sperm, increased sperm kinetic parameters (79)

There are very few studies that have looked at the effects of probiotic administration on male fertility not attributable to a possible genitourinary infection. In a study with zebrafish, Valcarce et al. demonstrated that supplementation with probiotic species *Lactobacillus rhamnosus* and *Bifidobacterium longum* was associated with increased sperm concentration, total motility, and progressive sperm motility in the treatment group (77). The two probiotic species also had known antioxidant properties, potentially highlighting their mechanism of action in male infertility (68). In a small pilot study of nine men with asthenozoospermia treated with the same oral antioxidant probiotic strains, sperm motility was drastically improved after 6 weeks of ingestion (74). DNA fragmentation and intracellular hydrogen peroxide were also decreased in sperm (74). Cell viability was not affected by the treatment (74). In a randomized controlled trial conducted by Maretti and Cavallini, the administration of a probiotic and prebiotic combination for six months was significantly associated with improved sperm count, ejaculate volume, sperm concentration, progressive motility and the progression of typical forms (75). The study also found the treatment to be significantly associated with an increased FSH, LH, and T levels in patients, with five men fathering a child compared to none in the control group. Another randomized control trial by Helli et al. looked at probiotic supplementation in men with idiopathic oligoasthenoteratozoospermia over a course of 10 weeks (76). In the treatment group, the ejaculate volume, number, concentration and percentage of motile sperm, and total antioxidant capacity of plasma significantly increased compared to the control, and the concentration of inflammatory markers significantly decreased (76). Probiotic supplementation led to a significant increase in sperm concentration and motility with a significant reduction in oxidative stress and inflammatory markers (76). The data from these studies is promising, but larger studies will be needed to fully understand the impact of probiotic supplementation.

## Conclusion

The Human Microbiome Project (HMP) has revealed that microbiota exist in nearly every anatomical niche in the body and play a role beyond infection. An individual's microbiome is unique and changes throughout a lifetime and is influenced by factors such as genetics, diet, drugs, body size, surrounding environment, and hygiene. The gut and male reproductive system are interdependent through the microbiome, and this axis influences male reproductive health and infertility. Several studies have shown that the GM can influence testosterone levels and sperm production, modulate intestinal and androgen metabolism, and affect testicular function. Dysbiosis in the gut and reproductive system may lead to an increase in ROS and may contribute to male infertility. Treatment with probiotics offers an exciting opportunity to potentially correct microbiome dysregulation, but larger human studies are needed to fully examine this question.
